# Repurposing Cholinesterase Inhibitors as Antidepressants? Dose and Stress-Sensitivity May Be Critical to Opening Possibilities

**DOI:** 10.3389/fnbeh.2020.620119

**Published:** 2021-01-14

**Authors:** Paul J. Fitzgerald, Pho J. Hale, Anjesh Ghimire, Brendon O. Watson

**Affiliations:** Department of Psychiatry, University of Michigan, Ann Arbor, MI, United States

**Keywords:** acetylcholine, chronic stress, cholinesterase inhibitor, depression, antidepressant, u-shape, donepezil, physostigmine

## Abstract

When stress becomes chronic it can trigger lasting brain and behavioral changes including Major Depressive Disorder (MDD). There is conflicting evidence regarding whether acetylcholinesterase inhibitors (AChEIs) may have antidepressant properties. In a recent publication, we demonstrated a strong dose-dependency of the effect of AChEIs on antidepressant-related behavior in the mouse forced swim test: whereas the AChEI donepezil indeed promotes depression-like behavior at a high dose, it has antidepressant-like properties at lower doses in the same experiment. Our data therefore suggest a Janus-faced dose-response curve for donepezil in depression-related behavior. In this review, we investigate the mood-related properties of AChEIs in greater detail, focusing on both human and rodent studies. In fact, while there have been many studies showing pro-depressant activity by AChEIs and this is a major concept in the field, a variety of other studies in both humans and rodents show antidepressant effects. Our study was one of the first to systematically vary dose to include very low concentrations while measuring behavioral effects, potentially explaining the apparent disparate findings in the field. The possibility of antidepressant roles for AChEIs in rodents may provide hope for new depression treatments. Importantly, MDD is a psychosocial stress-linked disorder, and in rodents, stress is a major experimental manipulation for studying depression mechanisms, so an important future direction will be to determine the extent to which these depression-related effects are stress-sensitive. In sum, gaining a greater understanding of the potentially therapeutic mood-related effects of low dose AChEIs, both in rodent models and in human subjects, should be a prioritized topic in ongoing translational research.

## Introduction

It is well-established that chronic or marked acute psychological stress can induce Major Depressive Disorder (MDD) in susceptible individuals (Hosang et al., 2014; Bonde et al., 2016). The linkage between stress and depression is strong, and MDD can, at least in some cases, be considered a chronic stress response of the brain. However, we are only able to treat a subset of cases of MDD effectively, and so in order to ameliorate this burdensome neuropsychiatric disorder, we need new innovation. Contributing to the clinical heterogeneity of MDD, other factors besides marked stress or trauma, such as inflammation and immune dysregulation, may also take part in the etiology of MDD (Miller et al., 2009; Leonard, 2010; Lee and Giuliani, 2019).

Most of the drugs that are used clinically to treat MDD, such as the selective serotonin reuptake inhibitors (SSRIs), tricyclic antidepressants (TCAs), serotonin-norepinephrine reuptake inhibitors (SNRIs), and monoamine oxidase inhibitors (MAOIs) are thought to boost monoaminergic neurotransmitters: serotonin, norepinephrine, and dopamine. These classes of drugs have been extensively studied in rodent models that are experimentally exposed to chronic mild stress, to investigate their efficacy in reversing a depression-like state induced by stress (Brady, 1994; Svensson, 2000; Kasper and Hamon, 2009). There is, however, also a literature dating back decades on the mood-related properties of the neurotransmitter acetylcholine (which is not a monoamine) and pharmacological agents that act upon it (Janowsky et al., 1972a, 1983, 1994; Dulawa and Janowsky, 2018). While cholinergic drugs have not achieved significant clinical use for MDD treatment, scopolamine, which blocks muscarinic cholinergic receptors has been found to act as a rapid antidepressant in rodents as well as humans, and the cholinergic receptor agonist, nicotine, also has antidepressant-like properties (Dulawa and Janowsky, 2018; Duman, 2018). One possibility is that the antidepressant properties of TCAs are modulated by their anticholinergic characteristics (Mate et al., 2020). Additionally, the cholinergic system is well-known to relate to attention and degree of engagement in a task, and that subsequently is known to determine performance under acute stress (Stillman et al., 1997). Furthermore, it is known that acute stress induces lasting gene expression changes in the cholinergic system, with specific increases in the activity of acetylcholinesterase enzyme, reducing acetylcholine availability (Kaufer et al., 1998). However, the role of treatments aimed specifically at reversing this acetylcholinesterase increase, using acetylcholinesterase inhibitors (AChEIs) is insufficiently understood.

Here we will focus on AChEIs, which interfere with the synaptic degradation of acetylcholine, and which have also been studied to a limited degree in MDD or animal models of depression-like behavior. The mood-related or stress-relieving properties of AChEIs are not well-characterized, including with respect to counteracting the effects of acute or chronic stress (Dulawa and Janowsky, 2018). Several AChEIs are currently used worldwide to treat the neurological disorder, Alzheimer's disease (AD) and other dementias. Several studies have shown that clinical administration of AChEIs to individuals with mood disorders can accentuate depression, and in some cases attenuate mania or hypomania (Janowsky et al., 1972b; Carroll et al., 1973; Reynolds III et al., 2011), though some other studies indicate an antidepressant role for AChEIs in human subjects (Ago et al., 2011; Spalletta et al., 2013; Cummings et al., 2016; Akechi et al., 2017). This literature is discussed in further detail later in this publication. We point out here that little appears to be known about the mood-related properties of AChEIs in younger subjects not suffering from dementia or cognitive impairment.

As in humans, studies in animals have provided conflicting evidence on whether AChEIs promote only depression-like behavior or instead can in some cases have therapeutic properties. For example, a number of studies in rodents have suggested that the AChEI, physostigmine, acutely promotes depression-like behavior in the forced swim test (FST) or tail suspension test (Mineur et al., 2013; Addy et al., 2015; Van Enkhuizen et al., 2015). While on the other hand, several studies of other AChEIs find antidepressant-like effects in the FST or sucrose preference test, including upon chronic administration (Maurice et al., 2006; Islam et al., 2014; Papp et al., 2016). One way to reconcile these seemingly disparate findings is that the dose, as well as the chronicity of tests used, may play a major role in therapeutic outcome. In a recent study from our laboratory (Fitzgerald et al., 2020), we systematically studied the dose-behavior profile of donepezil, an AChEI, and found that dose was indeed key to determining antidepressant vs. pro-depressant effects. Specifically, a relatively standard laboratory dose of donepezil (2.0 mg/kg) promoted depression-like behavior in the C57BL/6J mouse FST, whereas two lower-concentration doses (0.2, 0.02 mg/kg) frequently had antidepressant-like properties (but not during the aversive first swim session). We also found that the therapeutic effects of low doses typically only emerged after two or more swim sessions, suggesting that the chronicity of the dosing matters.

In this publication, we review the human and rodent literature on the mood-related properties of AChEIs in an effort to further investigate the prospects of these drugs as antidepressant agents. Although the cholinergic-adrenergic hypothesis of mania and depression was put forth nearly 50 years ago (Janowsky et al., 1972a), the field of research into the mood-related effects of AChEIs is still in its infancy, with the exception of some seminal studies that have already been carried out (Mineur et al., 2013; Islam et al., 2014; Addy et al., 2015; Van Enkhuizen et al., 2015; Papp et al., 2016). Given that MDD remains a major public health problem worldwide, and that many individuals with mood disorders are resistant to existing pharmacotherapeutics (Nemeroff, 2007; Bachmann, 2018; Hasin et al., 2018), there is great urgency to further investigate under which conditions AChEIs exhibit mood elevating (or depressing) properties, including as a function of dose and exposure to acute or chronic psychosocial stress.

## The Cholinergic-Adrenergic Hypothesis

In 1972, Janowsky and colleagues put forth the cholinergic-adrenergic hypothesis of depression and mania, which posits that a low ratio of cholinergic to adrenergic signaling promotes mania, whereas a high ratio promotes depression (Janowsky et al., 1972a). In other words, high cholinergic tone, relative to noradrenergic tone, has been hypothesized to promote depression. Research since then, both in humans and in animal models, has tended to support this hypothesis. For example, studies of the AChEI physostigmine have shown that this drug increases immobility in the FST or tail suspension test in mice (Mineur et al., 2013, 2015, 2018a; Addy et al., 2015), a result that has been corroborated by others (Van Enkhuizen et al., 2015). Consistent with the cholinergic-adrenergic hypothesis of mood disorders, clinical administration of AChEIs to individuals with mood disorders has shown depression-promoting effects, and in some cases attenuation of mania or hypomania (Janowsky et al., 1972b; Carroll et al., 1973; Reynolds III et al., 2011). Also, the cholinergic muscarinic antagonist drug, scopolamine, has rapidly acting antidepressant properties in humans and animal models (Dulawa and Janowsky, 2018; Duman, 2018). This body of studies that are relevant to and support the cholinergic-adrenergic hypothesis is further discussed below.

## Rat Models of Putatively Enhanced Cholinergic Transmission

A number of studies have investigated whether supra-normal cholinergic signaling in rat models promotes depression-related behavior. For example, in a potential model of depression-like behavior, the AChEI physostigmine can induce alterations in plasma hormonal signaling in rats that parallel findings in humans with MDD (Downs et al., 1986). A number of other studies have addressed the so-called “Flinders” rats, which were selectively bred to either be sensitive (Flinders sensitive line; FSL) or resistant (Flinders resistant line; FRL) to depression-like factors. Their relative difference in sensitivity has been validated across a range of behaviors and stressors, and in fact their initial breeding was based on differential sensitivity to the depression-like behavioral effects of the irreversible AChEI and insecticide, diisopropyl fluorophosphate (DFP) (Overstreet, 1986; Overstreet et al., 1986; Kanemaru and Diksic, 2009). The FSL rats, relative to FRL, show for example greater immobility in the FST after injection of the muscarinic agonist, arecoline (Overstreet, 1986), or a greater degree of FST immobility after a mild footshock stressor (which is not a chronic stressor) (Overstreet et al., 1986). Further supporting the link between these Flinders lines and cholinergic systems is that FSL rats also show greater amounts of baseline rapid eye movement (REM) sleep (i.e., in the absence of chronic stress), consistent with hypercholinergic tone, given the role of acetylcholine in promoting REM sleep, plus the shorter REM latency in human subjects with MDD (Benca et al., 1996). FSL rats, relative to FRL, also show exaggerated hypothermic responses to the AChEI physostigmine, consistent with a depression-like phenotype, but similar thresholds to rewarding electrical brain stimulation (Matthews et al., 1996).

However, another study of Flinders rats suggests chronic treatment of DFP at doses known to downregulate muscarinic receptors does not alter FST immobility in either FSL or FRL animals. This study went on to investigate several other pharmacological agents and concluded that Flinders rats may be a model of depression-like behavior through serotonergic or noradrenergic, rather than cholinergic, mechanisms (Schiller et al., 1992). Regardless of the endogenous cholinergic characteristics of Flinders rats, these studies implicate AChEIs, and acetylcholine, in the modulation of mood-related behavior, perhaps especially that high levels of cholinergic signaling promote depression-like behavior.

## Organophosphate Toxicity Studies

Organophosphate pesticides or nerve agents—including DFP, chlorpyrifos, paraoxon, soman, malathion—act as irreversible inhibitors of the enzyme, acetylcholinesterase. These drugs stand in contrast to reversible AChEIs, such as donepezil, rivastigmine, galantamine, and physostigmine, that are commonly used clinically to treat dementia or glaucoma. The acetylcholinesterase enzyme serves to reduce synaptic levels of acetylcholine and inhibiting it increases synaptic acetylcholine. A number of rodent studies have found that irreversible AChEIs increase immobility in a key rodent test for depression-related behavior, the FST, both in the case of acute administration (Wright et al., 2010; Deshpande et al., 2014; Pan et al., 2015) or repeated dosing (Ramos et al., 2006; Brocardo et al., 2007; Chen et al., 2011, 2012, 2014; Phillips and Deshpande, 2016). Ramos et al. (2006) did not, however, find that acute administration of malathion affected immobility time in the FST (Ramos et al., 2006). These drugs can also modulate locomotor- and anxiety-related behavior (Deshpande et al., 2014; Pan et al., 2015; Phillips and Deshpande, 2016). In summary, while these studies typically used high doses of irreversible AChEIs, they are consistent with the depression-like facilitation predicted by the cholinergic-adrenergic hypothesis of mood disorders, and are also consistent with the effects of relatively high dose (2.0 mg/kg) donepezil we observed in the FST in our study (Fitzgerald et al., 2020).

## Depression-Like Effects in Rodent Forced Swim and Sucrose Preference Tests

There are a number of rodent studies indicating that reversibly-binding AChEIs, particularly physostigmine, promote depression-like behavior in the FST and related tests. Many of these studies have been conducted in mice. In REM sleep-deprived mice, physostigmine blocked the antidepressant-like increase in swimming behavior produced by the antipsychotic drug, clozapine (Asakura et al., 1995). Physostigmine has also been shown to potentiate the immobility enhancing effects of chronic social defeat stress in the FST, and amplify the decrease in social interaction time produced by this stressor, while not modulating sucrose preference (Fernandes et al., 2018).

A series of studies carried out by another group in mice has further implicated physostigmine in promoting depression-like behavior in the FST or tail suspension test (and in some cases, anxiety-like behavior). These behavioral effects of physostigmine (up to 0.5 mg/kg) can be reversed by muscarinic or nicotinic antagonist drugs, further implicating cholinergic mechanisms in this drug's depression-related effects (Mineur et al., 2013). This study also showed that a decrease in hippocampal acetylcholinesterase activity, including when this is carried out pharmacologically, can decrease resilience to chronic social defeat stress (Mineur et al., 2013). Other studies by this group implicate β2 or α7 nicotinic acetylcholine receptor subunits in the amygdala or hippocampus, based on pharmacological modulation with the non-selective nicotinic antagonist mecamylamine or viral-mediated receptor downregulation experiments, in conferring some of the depression-related behavioral effects of cholinergic signaling (Mineur et al., 2016, 2018b). Cholinergic-noradrenergic interaction may modulate the observed depression-related behavior (Mineur et al., 2018a), and these studies also suggest that there are sex differences in the effects of cholinergic signaling on mood-related behavior in mice (Mineur et al., 2015, 2018b). The noradrenergic α2A agonist drug, guanfacine, was shown to reverse depression-like behavior in the FST that was induced by physostigmine (Mineur et al., 2015). This latter result may be surprising in light of the cholinergic-adrenergic hypothesis, given that guanfacine *lowers* presynaptic release of norepinephrine, which might be expected to potentiate the depression-like effects of physostigmine. But perhaps the agonist activity of guanfacine on postsynaptic α2A receptors facilitates noradrenergic signaling. The depression-related effects of physostigmine in mice, reported in this paragraph, have been corroborated by another group at a low dose (0.03 mg/kg) (Van Enkhuizen et al., 2015).

Additional studies in rats further implicate physostigmine in promoting depression-like behavior. Administration of physostigmine to rats that were exposed to inescapable footshooks, delivered intermittently over a 60 min period the day before the FST, amplified the decrease in active behaviors in the FST that was produced by this stressor (Płaznik et al., 1988). It has also been shown in rats that infusion of physostigmine into the ventral tegmental area (or systemic administration of low doses), increases immobility in the FST, suggesting that dysregulation of reward-related brain circuits may in part mediate the effects (Addy et al., 2015; Small et al., 2016). The therapeutic-like effects of the antidepressants, desipramine and nomifensine, in the FST can be antagonized by physostigmine (Mancinelli et al., 1988), consistent with the cholinergic-adrenergic hypothesis. An additional rat study, measuring anhedonia-related behavior through intracranial self-stimulation, found that another AChEI, donepezil, did not modulate the effects of chronic social defeat stress on this behavior (Gottschalk et al., 2018). This latter study raises the point that it will be important to determine if the depression-like effects of physostigmine generalize to other AChEIs, although we have already observed depression-like effects with donepezil (Fitzgerald et al., 2020). In summary, the above studies of physostigmine in mice and rats strongly implicate this drug in promoting depression-like behavior at higher doses in the range of 0.125–2.0 mg/kg and sometimes at lower ones.

## Antidepressant-Like Findings in Rodent Behavioral Tests

In contrast to the literature reviewed above, there are several studies, in addition to our own on donepezil, that report antidepressant-like effects of AChEIs in rodents. A study of swiss mice in the FST reported acute antidepressant-like properties of donepezil, including at very high doses (up to 30 mg/kg) (Maurice et al., 2006). This group also found that the AChEIs rivastigmine and tacrine lacked an antidepressant-like response, and they hypothesized that the antidepressant-like response of donepezil was not mediated through cholinergic mechanisms but rather involved the sigma-1 receptor. Maurice et al. (2006) also did not find a depression-like response to donepezil at high doses, and therefore did not suggest a Janus-faced dose-response pattern. One possibility is that swiss mice do not respond similarly to donepezil in the FST as the C57BL/6J strain we used in Fitzgerald et al. (2020). A study that used chronic treatment with the AChEI, rivastigmine, in olfactory bulbectomized mice (of the DDY strain), which is a rodent model of depression, reported antidepressant-like behavior in various tests including the FST, tail suspension test, and novelty suppressed feeding test (Islam et al., 2014). These authors suggest that the therapeutic-like effects of rivastigmine are dependent upon signaling of the serotonergic 5-HT_1A_ receptor.

While the effects of AChEIs on depression-like behavior that has been induced in rodents through exposure to chronic stress, remain poorly characterized at this time, they have been investigated in a fairly recent study. Those investigators found that in rats exposed to 8 weeks of chronic mild stress and treated chronically with the AChEIs rivastigmine or donepezil, there was an antidepressant-like response to these agents in the sucrose preference test (Papp et al., 2016). In that study, the therapeutic dose of donepezil (0.3 mg/kg) is similar to one of the doses in our study (0.2 mg/kg) that frequently exhibited antidepressant-like effects in mice (Fitzgerald et al., 2020).

Other rodent studies have found antidepressant-like behavioral effects with AChEIs, or putative AChEIs, that are less frequently studied. These AChEI drugs, plus the reversible ones described above, encompass a broad range of molecular structures and yet share antidepressant-like properties, implicating acetylcholine as a likely mediator of these therapeutic effects, instead of another single off-target pathway (see below). Chronic administration of two putative AChEIs, Huperzine A and components of rosemary tea, has been found to produce antidepressant-like effects in mice and rats, suggesting that phytochemical AChEIs may possess therapeutic properties in mood-related behavior (Ferlemi et al., 2015; Du et al., 2017). The AChEI ladostigil (TV-3326), when administered chronically, possesses antidepressant-like properties in the rat FST, but this may be due to additional monoamine oxidase inhibition (Weinstock et al., 2002; Poltyrev et al., 2005).

In summary, there are several studies that report antidepressant-like properties for AChEIs, but some of the authors have suggested these are due to non-cholinergic mechanisms of action. However, the wider the range of molecules that are known to inhibit acetylcholinesterase and that also possess antidepressant-like behavioral properties in rodents, the more likely it is that at least some of these drugs are acting through cholinergic mechanisms instead of another specific target.

## Janus-Faced or U-Shaped Dose-Response Curves

One way to reconcile the conflicting data reported above on the depression-related effects of AChEIs in rodents, is that these drugs may possess Janus-faced dose-response characteristics, where relatively low doses are therapeutic-like and high doses are aversive or depression-like. (We suggest that the term “Janus-faced” is a more accurate description of the mood-related effects of AChEIs than a “u-shaped” dose-response curve, since the latter term usually implies that the therapeutic effects of a low dose merely disappear at a high dose, rather than become aversive or toxic. When briefly describing some previous studies here, however, we will use both terms due to uncertainty over which term is more scientifically correct.) There is already a literature suggesting that neuromodulators such as norepinephrine, dopamine, serotonin, and acetylcholine exhibit Janus-faced or u-shaped dose-response curves for a range of behaviors (Baldi and Bucherelli, 2005). It has been suggested, based on rodent and monkey data, that “optimal” concentrations of synaptic norepinephrine and dopamine, particularly in prefrontal circuits, promote healthy cognition, including working memory (Arnsten, 2007; Vijayraghavan et al., 2007). Other studies of norepinephrine in rodents suggest that varying amounts of noradrenergic signaling shift the balance of prefrontal vs. amygdala functionality to modulate fear conditioning and extinction, also suggesting Janus-faced or u-shaped dose-response qualities (Giustino et al., 2016; Giustino and Maren, 2018). It has also recently been shown that the serotonergic 5-HT_1A_ receptor may regulate impulsive responding in rodents in a biphasic dose-response manner (Groft et al., 2019).

While there does not appear to be a large literature on mood-related Janus-faced or u-shaped dose-response properties of AChEIs, there are several learning and memory studies in rodents on this topic. In a study that lesioned the cholinergic nucleus basalis in rats, physostigmine improved performance in a water maze task at low doses while impairing it at a higher one (Mandel and Thal, 1988). Two rat studies of scopolamine-induced amnesia in the 8-arm radial maze found that several AChEIs, including physostigmine, show Janus-faced or u-shaped response characteristics, with lower doses reversing the amnesia and the highest ones restoring it (Braida et al., 1996, 1998). Another rat study that investigated nucleus basalis lesions as well as scopolamine-induced amnesia, found that several AChEIs exhibited therapeutic properties at lower doses while not doing so at higher doses (Wanibuchi et al., 1994). In a mouse active avoidance task, intraventricular infusion of physostigmine improved memory retention at low doses while not improving it at higher ones (Flood et al., 1981). It has also been proposed that acetylcholine exhibits Janus-faced or u-shaped dose-response characteristics with respect to drug seeking behavior or natural reinforcers (Grasing, 2016). If low dose AChEIs have therapeutic effects on learning and memory in rodents, this may be consistent with such restricted doses also improving mood-related behavior. For the above two rat studies that used systemic injections of physostigmine (Mandel and Thal, 1988; Braida et al., 1996), the therapeutic dosage range was 0.06–0.5 mg/kg, and the impairing or non-therapeutic range was 0.32–1.0 mg/kg. Recall that in the several physostigmine studies reviewed above that induced depression-like behavior in mice and rats, the dosage range was 0.125–2.0 mg/kg, which traverses the non-therapeutic range of the previous two papers on learning and memory, but also partially overlaps with their therapeutic range. This may suggest some degree of correlation between the affective-like and cognitive-related dosage range of this drug, where both effects of physostigmine may be characterized by a shared Janus-faced or u-shaped dose-response curve.

**Figure 1:**
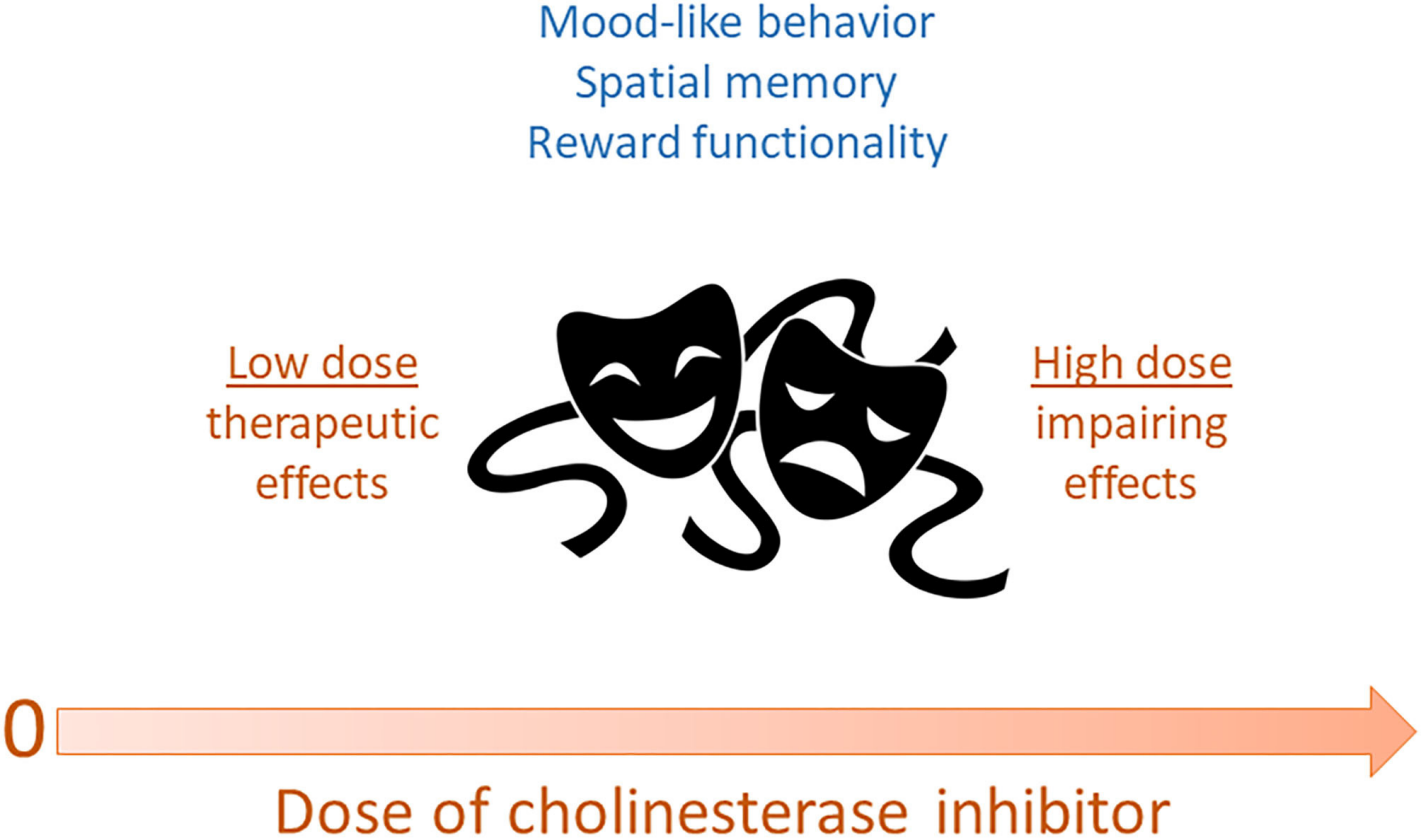
Dose of acetylcholinesterase inhibitors may exhibit a Janus-faced relationship with various functions. In this scenario, low doses of reversible cholinesterase inhibitors may produce optimal mood-like behavior, spatial memory, and reward functionality. In contrast, high doses of these drugs may impair these functions and have aversive effects. By extension, acetylcholine itself may be characterized by these same Janus-faced dose-response properties.

Our study (Fitzgerald et al., 2020) is the first, to our knowledge, to directly explore a potential Janus-faced or u-shaped curve in the behavioral effects of an AChEI as an antidepressant. Based on the previous literature, we systematically varied the dose of donepezil from 0.02–0.2 to 2.0 mg/kg in C57BL/6J mice and found that while the higher dose (2.0 mg/kg) recapitulated the previously-reported depressant-like properties in rodents, the lower doses (0.02 and 0.2 mg/kg) produced the antidepressant-like responses reported in other studies. Our data on donepezil also suggest that associative learning processes gate the emergence of antidepressant-related effects, as we typically did not observe therapeutic effects on the first FST exposure, or when the mice received the same dose of drug as they had during the first (and presumably most aversive) FST exposure. This is a topic that should be investigated further in future studies. In sum, our work nonetheless already supports a Janus-faced dose-response curve for AChEIs and also indicates that if used in a specified and controlled manner, AChEI medications may have antidepressant potential. Figure 1 summarizes the various data described above, suggesting that AChEIs have a Janus-faced dose-response curve for mood-related (and possibly other) functions.

## Stress and the Cholinergic System

While there does not appear to be a large literature on the interaction of psychosocial stress with affective-like behavioral responses to cholinergic drugs (including AChEIs), there are a number of studies on the interaction of stress with cholinergic signaling (e.g., Del Arco et al., 2007), where a recent one addresses how variable stress affects attentional processing (Eck et al., 2020). Microdialysis studies in freely moving rodents suggest that acute and even chronic stressors, such as restraint and footshock stress, can boost extracellular acetylcholine in medial prefrontal cortex and hippocampus, brain regions that are known to modulate mood-related behavior (Imperato et al., 1991; Mark et al., 1996; Mizoguchi et al., 2001). There is also theoretical and empirical evidence that either too much or too little cholinergic signaling can impair attentional functioning, consistent with a u-shaped or Janus-faced dose-response curve for this capacity (Perry and Perry, 1995; Sarter and Bruno, 1998). Rodent data have however suggested that prefrontal acetylcholine depletion does not affect attentional set formation or set shifting, but does impair serial reversal learning (Roberts et al., 1990, 1992; Robbins and Roberts, 2007). It has been suggested that whereas stress-enhanced cholinergic release can be adaptive behaviorally, chronic elevation of acetylcholine may lead to psychopathology such as MDD (Higley and Picciotto, 2014). An additional hypothesis put forth by this group is that high levels of cholinergic signaling may enhance encoding of stressful events, leading to negative encoding bias that is a principal feature of MDD (Mineur and Picciotto, 2019). Future preclinical and human subjects studies will be needed to better understand the complex interplay between cholinergic signaling, attentional processing, and psychosocial stress.

Since MDD can be induced in human subjects more significantly by chronic stress or trauma than by a single acute stressor of minor magnitude, this review has focused on how AChEIs may counteract the deleterious effects of chronic stress. Acute stress can increase synaptic release of acetylcholine and have an alerting effect, whereas severe or prolonged stress may elevate cholinergic signaling to the extent that it has a variety of negative consequences for the organism including increased depression-like behavior, as part of an Janus-faced curve (Mineur and Picciotto, 2019). It is noteworthy that many of the rodent behavioral assays we have been discussing (including in the context of chronic stress), such as the FST, are themselves acute stressors that presumably modulate cholinergic signaling. So while we know that acute stress can tune brain networks via cholinergic tone acutely affecting circuit tone, we do not yet have a mechanistic understanding about how the effects of chronic stress are altered by cholinergic medications such as AChEIs.

## Stress-Sensitivity of AChEI Effects

The large majority of animal studies of AChEIs that are reviewed above did not explore the effects of these drugs in the context of chronic mild stress. Since MDD in human subjects is a stress-linked disorder (Hosang et al., 2014; Bonde et al., 2016), it will be important to further investigate if chronic stress modulates the therapeutic effects of AChEIs in rodents. A recent study from our lab demonstrated that another novel antidepressant, ketamine, is only antidepressant-like in the mouse FST after exposure to unpredictable chronic stress (Fitzgerald et al., 2019), which parallels findings in humans that ketamine is therapeutic in subjects with depression, whereas it induces transient depression in healthy controls (Nugent et al., 2018). Whether AChEIs exhibit stress-sensitivity in rodent models is not clear. Several chronic social defeat studies reviewed above found that AChEIs only appear to have stronger depression-like effects after this stressor (Mineur et al., 2013; Fernandes et al., 2018), or are without effect (Gottschalk et al., 2018). Papp et al. (2016) in contrast showed in rats that rivastigmine and donepezil counteract the effects of chronic mild stress on sucrose preference (Papp et al., 2016). Antidepressant-like effects of the AChEI, Huperzine A, have also been reported in rats exposed to unpredictable chronic stress in a model of post-stroke depression (Du et al., 2017). Future studies in animal models (and human subjects) should more thoroughly investigate the extent to which it is necessary for the subject to be in a stress-induced depression-like state, for AChEIs to exhibit therapeutic properties.

## Human Subjects Research

Perhaps surprisingly, there are already a number of publications suggesting antidepressant properties of AChEIs in human subjects suffering from Alzheimer's disease (AD). MDD and AD are often comorbid, potentially due to shared underlying pathophysiological mechanisms (Ciaramella et al., 2016), consistent with AChEIs being therapeutic in both disorders. Specifically, there is degeneration of the cholinergic basal forebrain in AD, which may reduce cholinergic tone, and this reduced tone may contribute to overall brain neuromodulation including that needed for mood regulation. Relatedly, it has been noted that MDD in AD is qualitatively different in some regard from depression in cognitively healthy and/or younger individuals, with AD-MDD individuals having more prominent motivational symptoms and less pronounced affective symptoms than MDD-alone individuals (Hsiao and Teng, 2013).

Whereas some studies find that AChEIs do not affect depressive symptomatology in geriatric MDD without AD (Reynolds III et al., 2011; McDermott and Gray, 2012; Devanand et al., 2018) or in individuals with AD or related dementias (Vida et al., 1989; Rosenblatt et al., 2008; Jawaid et al., 2015; Ford and Almeida, 2017), a number of other studies report improvement of MDD or related measures accompanying dementia. Regarding improvement, a number of studies of AD (and in some cases, related dementia) have found that chronic administration of AChEIs such as donepezil, rivastigmine, and galantamine improve depressive symptomatology (Weiner et al., 2000; Tanaka et al., 2004; Mega et al., 2005; Rozzini et al., 2007; Ago et al., 2011; Pinto et al., 2011; Spalletta et al., 2013, 2014; Cummings et al., 2016; Akechi et al., 2017). Some findings on AChEIs suggest they may be of benefit for the depression-related symptoms of apathy and agitation in AD (Mega et al., 1999, 2005; Passmore et al., 2008; Pinto et al., 2011; Cummings et al., 2016). Also, discontinuation of AChEIs that had been used in dementia treatment can be associated with adverse behavioral changes such as decreased time engaging in leisure activities (Daiello et al., 2009). There is also a study reporting potential improvement of depression-related negative signs with AChEIs in schizophrenia (Stryjer et al., 2004), and a case report found improvement in treatment-resistant psychotic depression on these drugs (Smart et al., 2018).

In the studies summarized in the previous paragraph, examination of the doses of AChEIs used in the studies that do not vs. those that do report antidepressant-related effects does not reveal an obvious, systematic difference in the dosage range used. For example, a common dosage range for donepezil in human subjects appears to be 5–10 mg/day, both in positive (Weiner et al., 2000; Rozzini et al., 2007; Spalletta et al., 2014) and negative (Reynolds III et al., 2011; McDermott and Gray, 2012; Devanand et al., 2018) studies. It is interesting to note that if most adult human subjects weigh roughly 50–100 kg, this range of donepezil doses comprises 0.05–0.2 mg/kg/day, which is quite similar to the acutely therapeutic doses (0.02, 0.2 mg/kg) we observed with this drug in our mouse FST experiments (Fitzgerald et al., 2020). In other words, the commonly used dosage range for donepezil in humans may already be in the therapeutic range for treating mood-related symptoms.

In summary, there are mixed findings on the therapeutic effects of AChEIs on depression in elderly subjects who are suffering from AD, or in some studies of individuals who are cognitively healthy. There are nonetheless a number of studies reporting antidepressant properties. One potential source of variability in these studies is that different individuals with AD may exhibit variation in the underlying pathophysiology of the disease. It should also be noted that there appears to be a scarcity of studies on the mood-related effects of these drugs in younger, cognitively healthy individuals. It has already been suggested by others that AChEIs have u-shaped or Janus-faced dose-response properties in treating AD (Canal and Imbimbo, 1996; Braida and Sala, 2001; Calabrese, 2008), although their toxicity at high doses may make these studies more difficult to carry out. Future studies may more carefully address whether, in MDD with or without comorbid dementia, these drugs have u-shaped or Janus-faced properties within the relatively safe, non-toxic dosage range.

## AChEIs and Stressed Humans

There does not appear to be a large literature on the mood-related effects of AChEIs in humans who have been exposed to marked psychological stress or trauma, although there are studies on the potentially therapeutic properties of these drugs in post-traumatic stress disorder (PTSD). For example, rivastigmine may have efficacy as an adjunctive pharmacological agent in chronic PTSD (Fayyazi Bordbar and Talaei, 2013). Future studies should address in greater detail whether AChEIs have mood modulating properties in individuals who have been exposed to chronic stress or trauma and are exhibiting MDD or PTSD, with comparison to healthy controls.

## Limitations of Behavioral Despair Models

In recent years, rodent behavioral despair models such as the FST and tail suspension test, have been questioned as a translational model for human MDD (De Kloet and Molendijk, 2016; Commons et al., 2017; Molendijk and de Kloet, 2019). These commonly used assays are now thought to be largely measures of stress coping and how this is modulated by monoaminergic antidepressants, although stress coping in the FST is still arguably relevant to MDD (De Kloet and Molendijk, 2016; Commons et al., 2017). This reinterpretation of the FST in the last several years not only needs to be considered (in future studies) when exposing rodents to chronic stress and then measuring the putative therapeutic-like effects of drugs such as AChEIs, but also in modeling neuropsychiatric disorders other than MDD that are characterized by altered responses to stress (Commons et al., 2017). Also, many of the tests we use preclinically to study putative antidepressants, such as the FST, are of short duration and may not match the ongoing nature of exposure to chronic stress. For this reason, longer-term assays in animals should be developed to better test whether cholinergic pharmacological agents counteract the deleterious effects of chronic stress (Nollet et al., 2019). Finally, chronic stress can induce hyperactivity in the FST in mice, which may be confounded with the putatively therapeutic-like effects of pharmacological agents such as AChEIs (Strekalova et al., 2005; Bogdanova et al., 2013).

## Possible Mechanisms of Dose-Dependent Action

While it is known that stress can impact cholinergic signaling over long periods, the importance of that change is not yet known. However, it is clear that the cholinergic system impacts brain functioning in realtime and presumably at all times. Increased acetylcholine is associated with attention and improved cognition (Parikh and Bangasser, 2020; Venkatesan et al., 2020) as well as organized neural oscillations and firing patterns correlated with active and engaged behavior, such as exploration, learning or seeking of necessary resources (Buzsaki, 2002). To the best of our understanding this is based on projections of cholinergic neurons from the medial septum and basal forebrain more generally up to the hippocampus and cortex (Záborszky et al., 2018). Thus, these effects may be based on one particular cholinergic projection system; however other cholinergic projection systems exist throughout the brain such as projections of the pedunculopontine nucleus (French and Muthusamy, 2018) that may be differentially sensitive to AChEIs administered peripherally. If these nuclei have opposing effects, a Janus-faced curve could be created across varying doses, where at low doses one effect may be activated and at higher doses a second and opposing effect may counteract the first. Or alternatively, the Janus-faced curve we observed may be due to Janus-faced responses in each/every cholinergic signaling tract. A specific possibility is that “antidepressant” properties, similar to the active engagement and exploration behavior seen with natural cholinergic increase are related to physiologic-scales of acetylcholine tone, whereas “pro-depressant” properties could be due to supra-physiologic cholinergic tone. However this possibility is certainly yet to be explored in detail, although it could be investigated with microdialysis experiments in rodents (Johnson et al., 2012) or possibly optogenetic approaches using systematically varied degrees of stimulation of differing cell groups (Vandecasteele et al., 2014; Hersman et al., 2017; Cissé et al., 2018). An additional point is that, at the receptor level, AChEIs might produce a Janus-faced dose-response relationship through various degrees of synaptically boosted acetylcholine acting differentially at muscarinic vs. nicotinic receptors. Determining how AChEIs mechanistically produce their putatively therapeutic-like effects remains a topic for future investigation.

## AChEIs, Cholinergic Antagonists, and Nicotine

As described above, a limited number of studies have shown that AChEIs can exhibit antidepressant-like properties in rodents, as can the agonist drug nicotine (Tizabi et al., 1999; Andreasen and Redrobe, 2009; Villégier et al., 2010; Haj-Mirzaian et al., 2015). Paradoxically, it has also been demonstrated that the nicotinic *antagonist* mecamylamine can also do so, as well as the muscarinic antagonists atropine and scopolamine (Mancinelli et al., 1988; Andreasen and Redrobe, 2009; Duman, 2018). How do we reconcile these apparent discrepancies? One study has suggested, for example, that nicotine and mecamylamine may both be antidepressant-like in mice either through differential effects on serotonin and norepinephrine, or by nicotine resulting in desensitization of nicotinic receptors and thereby mimicking an antagonist drug (Andreasen and Redrobe, 2009). The antidepressant-like properties of the M1 muscarinic cholinergic antagonist, scopolamine (Duman, 2018), may be related to its blocking of this receptor (Burke, 1986), which could be functionally opposed to other muscarinic receptors. Another general possibility for all of these drugs: perhaps there is a “symmetric” Janus-faced relationship between cholinergic signaling and depression-like behavior, such that mild increases or decreases in signaling are antidepressant-like, whereas more marked increases or decreases are depression-like. Future animal studies should address these hypotheses experimentally, using pharmacological agents, optogenetics or chemogenetics, or other techniques.

## Conclusions

The studies reviewed above, consisting of both animal and human data, suggest that AChEIs possess both depression-promoting and antidepressant qualities under various experimental conditions. The Flinders rat studies, while not necessarily comprising a hypercholinergic model of depression-like behavior, still implicate acetylcholine in mood regulation. Toxicity studies of irreversible organophosphate AChEIs suggest that high levels of synaptic acetylcholine promote depression-like behavior in the FST. Studies of reversibly binding AChEIs, particularly physostigmine, also suggest that high levels of synaptic acetylcholine increase rodent depression-like behavior in the FST and other tests. There is, however, also a small literature in rodents that suggests AChEIs can have antidepressant-like effects under some conditions. Studies in elderly humans with AD also have reported antidepressant effects of AChEIs, with some data suggesting lack of therapeutic efficacy. We suggest here that these disparate findings in animals and humans may be largely reconciled by synaptic acetylcholine having a Janus-faced dose-response relationship with mood-related behavior in critical brain circuits, with relatively low levels of signaling generally being therapeutic and high levels promoting depression or depression-like behavior. We suggest that future studies should more thoroughly investigate, in rodents and humans, the potentially mood elevating properties of low dose reversible AChEIs, including after exposure to chronic psychosocial stress.

## Author Contributions

All authors listed have made a substantial, direct and intellectual contribution to the work, and approved it for publication.

## Conflict of Interest

The authors declare that the research was conducted in the absence of any commercial or financial relationships that could be construed as a potential conflict of interest.
